# A Critical Review of Statistical Methods for Twin Studies Relating Exposure to Early Life Health Conditions

**DOI:** 10.3390/ijerph182312696

**Published:** 2021-12-02

**Authors:** Salvatore Fasola, Laura Montalbano, Giovanna Cilluffo, Benjamin Cuer, Velia Malizia, Giuliana Ferrante, Isabella Annesi-Maesano, Stefania La Grutta

**Affiliations:** 1 Institute for Biomedical Research and Innovation, National Research Council, 90146 Palermo, Italy; laura.montalbano@irib.cnr.it (L.M.); giovanna.cilluffo@irib.cnr.it (G.C.); velia.malizia@irib.cnr.it (V.M.); stefania.lagrutta@irib.cnr.it (S.L.G.); 2 Institute Desbrest of Epidemiology and Public Health, Inserm and University of Montpellier, 34093 Montpellier, France; benjamin.cuer@inserm.fr (B.C.); isabella.annesi-maesano@inserm.fr (I.A.-M.); 3 Department of Surgical Sciences, Dentistry, Gynecology and Pediatrics, Pediatric Division, University of Verona, 37134 Verona, Italy; giuliana.ferrante@univr.it

**Keywords:** children, exposome, genome, health, statistical methods, twin data

## Abstract

When investigating disease etiology, twin data provide a unique opportunity to control for confounding and disentangling the role of the human genome and exposome. However, using appropriate statistical methods is fundamental for exploiting such potential. We aimed to critically review the statistical approaches used in twin studies relating exposure to early life health conditions. We searched PubMed, Scopus, Web of Science, and Embase (2011–2021). We identified 32 studies and nine classes of methods. Five were conditional approaches (within-pair analyses): additive-common-erratic (ACE) models (11 studies), generalized linear mixed models (GLMMs, five studies), generalized linear models (GLMs) with fixed pair effects (four studies), within-pair difference analyses (three studies), and paired-sample tests (two studies). Four were marginal approaches (unpaired analyses): generalized estimating equations (GEE) models (five studies), GLMs with cluster-robust standard errors (six studies), GLMs (one study), and independent-sample tests (one study). ACE models are suitable for assessing heritability but require adaptations for binary outcomes and repeated measurements. Conditional models can adjust by design for shared confounders, and GLMMs are suitable for repeated measurements. Marginal models may lead to invalid inference. By highlighting the strengths and limitations of commonly applied statistical methods, this review may be helpful for researchers using twin designs.

## 1. Introduction

Investigating disease etiology is one of the major goals in the practice of epidemiology. However, controlling for the totality of confounding factors is a big challenge for investigators aiming to unveil the causal effect of a given exposure on a given disease [1].

Twins share a large number of genes, prenatal, neonatal, and postnatal factors, and provide a unique opportunity to account for the human genome and exposome [2,3]. In pediatric populations, twin data are particularly useful to shed light on the origin of associations between fetal and early life exposures and the risk of later diseases. In particular, if paired (conditional) analyses confirm the associations found through unpaired (marginal) analyses, there is evidence of a causal pathway; conversely, if paired analyses do not confirm the associations, they are to be ascribed to shared factors such as the genome or maternal variables [4,5].

Twin data also allow considering the genome as the exposure of interest [6]. Monozygotic (MZ) twins share 100% of their genes, while dizygotic (DZ) twins share about 50% of their genes. Therefore, if MZ twins are more similar than DZ twins with regard to the outcome of interest, this is indicative of an effect of the genome on that outcome. Conversely, non-genetic variability is to be ascribed to the totality of shared and individual environmental exposures (the exposome) [7].

Using appropriate statistical methods is fundamental for exploiting the potential of twin data and carrying out valid statistical inference [8]. In 2005, Carlin et al. [9] critically reviewed the regression approaches for the analysis of twin data, showing that transferring standard methods to twin data is not straightforward, because they need to account for the paired structure of the data that induces correlation between observations. Moreover, a clear awareness of the assumptions underlying each method and the consequent parameter interpretation is fundamental [9]. Recently, Tan et al. [10] reviewed twin methods used in epigenetic studies and found that simplistic approaches (disregarding within-pair dependence and potential confounding effects), such as the unpaired *t*-test, are rare but still present in the literature, possibly undermining the validity of the evidence base. Similar works have not been performed with regard to the methods applied in published twin studies relating exposure to diagnosable diseases in a broader sense.

This research was motivated by the earlyFOOD (“Long-term impact of gestational and early-life dietary habits on infant gut immunity and disease risk”) project (EU-H2020 Era-Net HDHL-INTIMIC cofounded call “Interrelation of the Intestinal Microbiome, Diet and Health”). Human microbial colonization is a crucial phase in the early development of a child. Indeed, a growing body of literature has associated early-life imbalances of the gut microbiota with several diseases, especially immune-mediated, metabolic, and neurodevelopmental disorders [11]. Colonization begins at birth when the newborn is exposed to microbes of primarily maternal origin. Then, it continues to develop under the influence of maternal antibodies transferred through breastfeeding, dietary-related metabolites, and environmental factors. In this regard, disentangling the role of different exposures in the etiology of the aforementioned conditions is of particular importance. Therefore, we focused on studies assessing the effects of diet or other exposures on the risk of obesity, allergic and neurodevelopmental disorders, aiming to review the statistical approaches used and to summarize their strengths and limitations.

## 2. Materials and Methods

On 12 April 2021, we searched PubMed, Scopus, Web of Science, and Embase, using combinations of Medical Subject Headings, explosion searches, and free keywords. The full search strategy is reported in the Supplementary Material S1.

The inclusion criteria were: (1) presence of children aged 0–12 years among the participants; (2) presence of statistical analyses carried out exclusively on twin pairs; (3) assessment of diet or other exposures; (4) obesity, asthma, eczema, rhinitis, and neurodevelopmental disorders were among the study outcomes; (5) publication year from 2011 to 2021; and (6) English language. The exclusion criteria were: (1) out of topic; and (2) case reports, reviews, or abstracts. The studies identified in the four databases were combined and duplicates were removed. Three reviewers (S.F., L.M., and S.L.G.) screened the articles for relevance based on titles and abstracts. The same reviewers screened the full texts of potentially eligible articles. The following information was extracted from the included articles: first author and publication year; outcomes; exposures and confounders; study design (including size and age of the twin sample); statistical methods; and main results.

## 3. Results

**Figure 1:**
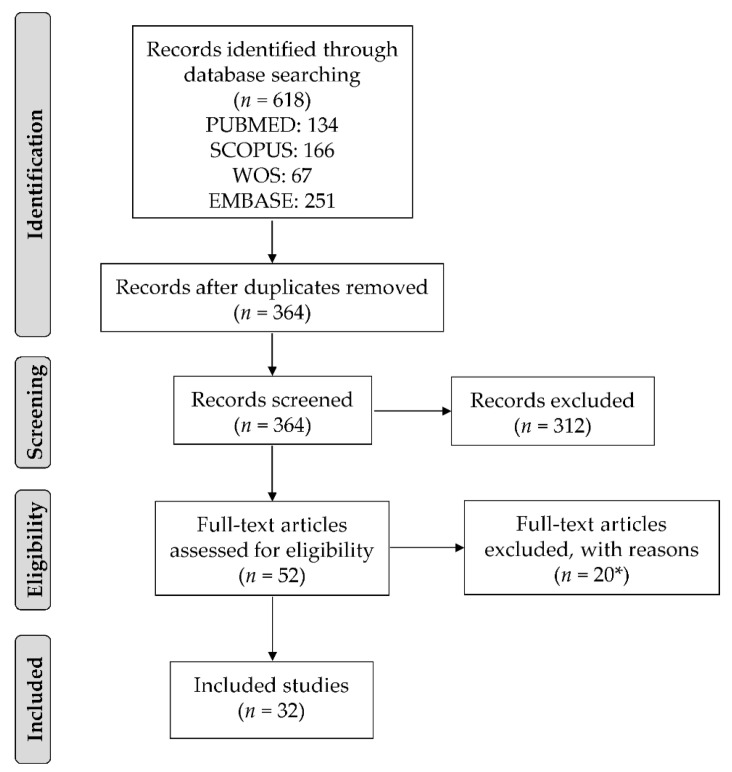
Flow diagram showing the study selection process. * Reasons for exclusion: reviews (*n* = 2), abstracts (*n* = 11), statistical analyses included singletons and twins together (*n* = 1), different outcomes (*n* = 3), twin mothers (*n* = 1), adult twins (*n* = 1), and no statistical analyses (*n* = 1).

Figure 1 summarizes the study selection process. We identified 618 articles through the four electronic databases. After the exclusion of duplicates, 364 articles were screened based on titles and abstracts, and 52 articles were identified as potentially eligible. Of them, 20 were excluded following full-text evaluation (Figure 1). Therefore, 32 articles were included in this review.

Table S1 summarizes the characteristics of the included studies. We identified nine classes of statistical methods. Thereafter, we will present them from the most complex (the most informative) to the most simple (the least informative) one, based on the number of model parameters and underlying assumptions. Five were conditional approaches (within-pair analyses): additive-common-erratic (ACE) models [12,13,14,15,16,17,18,19,20,21,22], generalized linear mixed models (GLMMs) [23,24,25,26,27], generalized linear models (GLMs) with fixed pair effects [28,29,30,31], within-pair difference analyses [32,33,34], and paired-sample tests [33,35]. Four were marginal approaches (unpaired analyses): generalized estimating equations (GEE) models [26,30,36,37,38], GLMs with cluster-robust standard errors [24,29,31,39,40,41], GLMs [42], and independent-sample tests [43].

### 3.1. ACE Models

A univariate ACE model [44] can be presented as:

(1) $$\{\begin{array}{c} Y_{1j}=\mu +aA_{1j}+cC_{1j}+eE_{1j}+\beta x_{1j}+\gamma z_{1j}\\ Y_{2j}=\mu +aA_{2j}+cC_{2j}+eE_{2j}+\beta x_{2j}+\gamma z_{2j} \end{array},$$

where $Y_{1j}$ and $Y_{2j}$ are quantitative outcome values in twins 1 and 2, respectively, with j=1,2,…,n and n equal to the number of twin pairs. Parameter μ is an intercept, x is an optional exposure of interest (β quantifies its effect), and z is an optional confounder (γ quantifies its effect); x and z may also be vectors.

The effect of genome and exposome is accounted for by means of unobserved random variables, encompassing additive genetic ($A_{1}$ and $A_{2}$), common (or shared) environmental ($C_{1}$ and $C_{2}$), and erratic (or unique) environmental ($E_{1}$ and $E_{2}$) factors. The random variables are assumed mutually independent within the same twin, and to follow a standard normal distribution. Consequently, $a^{2}$ is the outcome variance explained by genetic factors, $c^{2}$ is the variance explained by shared environmental factors, and $e^{2}$ is the variance explained by unique environmental factors (including measurement error). The percentage genetic contribution (heritability) can be derived as $a^{2}/\left(a^{2}+c^{2}+e^{2}\right)$; percentage environmental contributions can be derived similarly. The ACE model is identified due to the following assumptions: the correlation between $A_{1}$ and $A_{2}$ is 1 in MZ twins and 0.5 in DZ twins; the correlation between $C_{1}$ and $C_{2}$ is 1 ($C_{1}=C_{2}$) both in MZ and in DZ twins. All other pairs of latent variables are assumed independent between twins in the same pair. Model (1) can be estimated either from individual data or from observed sample sizes, means, and correlation matrices in MZ and DZ twins.

Only two studies applied model (1) in its plain form [12,14]. In such studies, the ACE model was applied to binary outcomes using tetrachoric correlations. In children aged 1 year, Bunyavanich et al. [12] highlighted a predominance of shared environmental contributions for asthma diagnosis and medication use (84 to 88% through different models) and a predominance of heritability for asthma hospitalizations (55 to 95%). In children aged 3 years, they highlighted a substantial genetic contribution for all three outcomes (34 to 65%). In children aged 3–9 years, Kahr et al. [14] highlighted an overall contribution of heritability equal to 94% for atopic dermatitis, 54% for asthma, and 43% for hay fever. Moreover, they did not find significant differences in the genetic contribution (gene–environment interactions) after stratification by age, sex, gestational age, delivery mode, and maternal smoking. In this regard, it appears that the authors applied a statistical test (e.g., a Wald test) to compare independent estimates obtained from the different sub-groups.

The moderated ACE model [45] offers a formal way of assessing gene–environment interactions by allowing parameters (including the intercept) to be linear functions of one or more moderators. Using this model in children aged 3 and 12 years, Robbers et al. [20] highlighted a lower heritability of internalizing/externalizing problems in children from divorced families (3 to 51%) than in children from non-divorced families (34 to 60%). In children aged 7, 10, and 12 years, Lamb et al. [16] found no shared environmental influences on internalizing/externalizing problems (quantitative scales). Moreover, they found lower heritability of internalizing/externalizing problems in twins exposed to different teachers (26 to 56%) than in twins exposed to the same teacher (67 to 82%). They also found higher heritability of externalizing problems in boys (47 to 82%) than in girls (55 to 75%). In children aged 4 years, Schrempft et al. [21] found that heritability of Body Mass Index (BMI) was significantly higher in higher-risk home environments (86%) than in lower-risk ones (39%). Moreover, they found a significantly lower effect of shared environmental factors on BMI in high obesogenic home environments (0%) than in low obesogenic home environments (34%). They also fit a scaled model allowing different sizes of variance components between moderator subgroups but fixing their percentage contributions to be equal. However, according to the likelihood-ratio tests performed, the scaled model generally led to a deterioration in the model fit.

When sex is the moderator, the gene–environment interaction is referred to as quantitative sex-limitation [46]. In this case, genetic and environmental effects on the outcome are assumed greater in one sex than in the other one. If, however, different subsets of genes (or shared environmental factors) are assumed to influence the outcome in boys and in girls, it is referred to as qualitative sex-limitation [46]. Clues of qualitative sex-limitation can be provided by a lower opposite-sex dizygotic (OSDZ) correlation. In this case, the correlation between $A_{1}$ and $A_{2}$ (or between $C_{1}$ and $C_{2}$) in the OSDZ group is expected to be <0.5 (or <1), and is estimated as a free parameter. Quantitative and qualitative sex-limitation models were applied by Silventoinen et al. [22], who found the lowest heritability of body mass index (BMI) at 4 years of age (40%) and increased heritability at 19 years of age (75%). Evidence of quantitative and qualitative (genetic) sex-limitation was found through likelihood-ratio tests carried out for each age (from 1 to 19 years): age-specific patterns of genetic/environmental contributions were similar in boys and girls, and genetic correlations within OSDZ pairs were generally <0.5.

An important extension of model (1) is the multivariate ACE model. Here we present the Cholesky decomposition representation of the bivariate case [47]:

(2) $$\{\begin{array}{c} Y_{1j}=\mu_{y}+a_{y}A_{1j}^{y}+c_{y}C_{1j}^{y}+e_{y}E_{1j}^{y}+\beta_{y}x_{1j}+\gamma_{y}z_{1j}\\ Y_{2j}=\mu_{y}+a_{y}A_{2j}^{y}+c_{y}C_{2j}^{y}+e_{y}E_{2j}^{y}+\beta_{y}x_{2j}+\gamma_{y}z_{2j}\\ W_{1j}=\mu_{w}+a_{yw}A_{1j}^{y}+c_{yw}C_{1j}^{y}+e_{yw}E_{1j}^{y}+a_{w}A_{1j}^{w}+c_{w}C_{1j}^{w}+e_{w}E_{1j}^{w}+\beta_{w}x_{1j}+\gamma_{w}z_{1j}\\ W_{2j}=\mu_{w}+a_{yw}A_{2j}^{y}+c_{yw}C_{2j}^{y}+e_{yw}E_{2j}^{y}+a_{w}A_{2j}^{w}+c_{w}C_{2j}^{w}+e_{w}E_{2j}^{w}+\beta_{w}x_{2j}+\gamma_{w}z_{2j} \end{array}$$

where Y and W are different quantitative outcomes, with specific intercepts and covariate effects. There are twice as many latent random variables as in model (1), and the assumptions about their variances and covariances remain similar. For W, the variance explained by heritable factors is now $a_{yw}^{2}+a_{w}^{2}$, and environmental contributions can be derived similarly. Two meaningful pieces of information can be derived from model (2). The first one is the fraction of the correlation (r) between Y and W that can be attributed to common genetic factors: $FR_{a}=a_{y}a_{yw}/\left(a_{y}a_{yw}+c_{y}c_{yw}+e_{y}e_{yw}\right)$. The second one is the correlation between genetic components of Y and W (i.e., the “genetic correlation”): $r_{a}=a_{y}a_{yw}/\sqrt{a_{y}^{2}\left(a_{yw}^{2}+a_{w}^{2}\right)}$. Similar indicators can be derived for environmental factors.

In children aged 4–7 years, Faith et al. [13] estimated heritability of BMI, waist circumference (WC), and per cent body fat (BF) to be 89%, 73%, and 90%, respectively. Then, a bivariate ACE model highlighted that energy compensation ability (COMPX) was correlated with BF (r=−0.24) due to a unique environmental correlation ($r_{e}=-0.27$). In twins aged 8–17 years, Ning et al. [19] found that heritability ranged from 56 to 71% for BMI and from 24 to 56% for WC, with substantial contributions of common genetic effects to correlations ($FR_{a}$) between BMI and WC (37 to 91%). They also found higher genetic effects in older children and in girls, but this appears to have been assessed through univariate models. Indeed, multivariate analysis of gene–environment interaction and sex-limitation is not straightforward [46]. Interestingly, Llewellyn et al. [17] applied a bivariate ACE model to repeated measurements of BMI in children at 4 and 10 years. They found that heritability increased significantly from 43 (age 4) to 82% (age 10), and that genetic correlation between BMI at the two ages was $r_{a}=0.58$.

The extension of model (2) to more than two outcomes requires including additional equation pairs with additional random variables (in a cumulative fashion), so that parameters like $a_{yw}$, $c_{yw}$ and $e_{yw}$ are obtained for each outcome pair. Using a multivariate ACE model in children aged 3 months, Llewellyn et al. [18] found weak correlations between weight and slowness in eating (r=0.22), satiety responsiveness (r=0.23), and appetite size (r=0.30), and that common genetic effects explained about 40% of these correlations. Finally, in children aged 5 years, Kan et al. [15] found high heritability of BMI (79%) and weak correlations between BMI and food responsiveness (r=0.20) and external eating (r=0.10). Since the different contributions to correlation ($FR_{a}$, $FR_{c}$, and $FR_{e}$) were in opposite directions, these were not reported by the authors.

In all the aforementioned studies, the whole genome (accounted for by random variables A) and the exposome (accounted for by random variables C and E) were themselves the exposures of interest (no study aimed to estimate parameter β for a specific exposure). The reported effects were in the form of a variance (or correlation) component and can be considered indicative of a causal pathway to the outcomes. This would be especially true as all the studies accounted for several confounders/effect modifiers by regressing them out prior to analyses, inclusion in the model, or stratification (Table S1).

### 3.2. GLMMs

A GLMM can be expressed as:

(3) $$g\left[E\left(Y_{ij}|b_{j}\right)\right]=\mu +b_{j}+\beta x_{ij}+\gamma z_{ij},$$

where g is a known link function that relates the expected value of the outcome (belonging to the exponential class of distributions) to the linear predictor, $Y_{ij}$ is the outcome value in twin i (i=1, 2) of pair j, and $b_{j}$ is a pair-level random effect, generally assumed to be normally distributed with 0 mean and variance equal to $\sigma_{b}^{2}$. Interpretation of β and γ changes according to whether g is identity (mean difference), logarithmic (log-relative risk, RR), or logistic (log-odds ratio, OR). Using model (3), Pimpin et al. [27] found that daily % of total energy from proteins (%Epro) at 21 months was associated with overweight/obesity status at 36 months (OR = 1.10). Similarly, Pimpin et al. [25] found that % dairy protein intake at 21 months was associated with overweight/obesity status at 36 months (OR = 1.12). Ha et al. [23] found that postnatal exposures to O_3_ were significantly associated with developmental delays at 8, 12, 18, 24, 30, and 36 months (RR = 1.014 to 1.173). Moreover, they found that postnatal exposures to PM_2.5_ were significantly associated with personal-social developmental delays up to 18 months (RR = 1.052 to 1.120). Finally, model (3) was used by Jackson et al. [24] to confirm the results obtained through a different method.

An extension of model (3) suitable for repeated measurements (e.g., for growth models) can be obtained by adding an individual-level random intercept and, possibly, a random slope for the time indicator, with a structure of variance/covariance between them. Using this extension, Pimpin et al. [27] found that %Epro was associated with BMI (β=0.043) and weight (β=0.052) in children aged between 21 and 60 months. Similarly, Pimpin et al. [25] found that % dairy protein intake was associated with BMI (β=0.040) and weight (β=0.046). Yeung et al. [26] found that infertility treatments were not associated with developmental delays in children aged between 4 and 36 months. Finally, Ha et al. [23] found that prenatal exposure to O_3_ was significantly associated with communication delays (RR = 1.025) between 8 and 36 months.

Since GLMMs include pair effects, shared confounders are adjusted for by design. Therefore, the effects sizes reported in the aforementioned studies may be considered indicative of a causal pathway from the exposures of interest to the outcomes. This would be especially true as all the studies explicitly accounted for several confounders (both shared and individual-level ones) by inclusion in the model (Table S1).

### 3.3. GLMs with Fixed Pair Effects

A GLM with fixed pair effects ($b_{j}$) can be defined as:

(4) $$g\left[E\left(Y_{ij}\right)\right]=b_{j}+\beta x_{ij}+\gamma z_{ij}.$$

In this case, parameters $b_{j}$ are estimated through dummy variables. Using this model in twins aged 9–16 years, Örtqvist et al. [29] found no association between fetal growth and lung function. In children aged 4 years, Leong et al. [31] found no association between the number of antibiotic courses during early childhood (0–24 months) and BMI/obesity status. For binary outcomes, conditional logistic regression allows eliminating the need to estimate the strata parameters $b_{j}$. Through this model, Örtqvist et al. [29] found a strong association between fetal growth and abnormal FEV_1_ (OR = 5.57) in twins aged 9–16 years. In children up to 18 years, Li et al. [28] found an increased risk of ever being obese associated with untreated infection in the first year of life compared with no infection (OR = 1.55). Finally, in children aged 3–10 years, Slob et al. [30] found that antibiotic use in the first 2 years of life was consistently associated with an increased risk of asthma in two cohorts (OR = 1.54 and OR = 2.00), while inconsistent results were found in the two cohorts for eczema (OR = 0.99 and OR = 1.67).

Since this class of models includes pair effects, the effects sizes reported in the aforementioned studies may be considered indicative of a causal pathway from the exposure to the outcome. This would be especially true in two of the four aforementioned studies where several individual-level confounders were included in the model [30,31]. However, it should be pointed out that in one study [31], model (4) was applied to a binary outcome using a standard logistic regression with dummy variables for the pair effects. In this case, the estimator of β is severely biased, and using conditional logistic regression is much more recommended (see Discussion).

### 3.4. Within-Pair Difference Analyses

When g is the identity function, a within-pair difference model can be derived from (and is formally equivalent to) model (4) by taking differences between twins 1 and 2:

(5) $$E\left(Y_{1j}-Y_{2j}\right)=\beta \left(x_{1j}-x_{2j}\right)+\gamma \left(z_{1j}-z_{2j}\right).$$

This model was applied by Bogl et al. [32] in twins aged 11 years or older, who found that increasing energy intake was associated with increasing BMI (β=0.13 for a 100-kcal/d increase). Of note, other authors simply analyzed correlations between outcome and exposure differences. In children aged 3 months, Tripicchio et al. [34] used Pearson’s correlation and found that parental restriction to eat was positively associated with BMI (r=0.31), while it was negatively associated with an energy compensation ability scale (COMPX, r=−0.27). Moreover, parental pressure to eat was negatively associated with BMI (r=−0.40), BF (r=−0.38), and WC (r=−0.40). Dubois et al. [33] used Spearman’s correlation and found negative (positive) associations between intake of carbohydrates (fats) at 9 years and BMI at 12, 13, and 14 years.

As for model (4), parameter β in model (5) represents a within-pair effect of the exposure, and may be considered indicative of a causal pathway to the outcome. However, only one study [32] included an individual-level confounder (height) in the model.

### 3.5. Paired-Sample Tests

If all the twin pairs are discordant on a binary exposure ($x_{1j}=1$ and $x_{2j}=0$), a paired-sample test can be applied. Particularly for a quantitative outcome, and in the absence of individual-level confounders (γ=0), performing a paired *t*-test is equivalent to testing β in model (5), which becomes:

(6) $$E\left(Y_{1j}-Y_{2j}\right)=\beta .$$

Similarly, a paired-sample test can be performed if all the twin pairs are discordant on a binary outcome. Through Wilcoxon signed-rank tests, Dubois et al. [33] found that heavier female twins consumed more grain products than their leaner female co-twin. 

Moreover, in twins aged 4–18 years, Bilenberg et al. [35] found that twins scoring high on a scale of attention-deficit/hyperactivity disorders (ADHD) had significantly higher PnPs14 RFU-score (a maternal transplacentally-acquired antibody) than lower-scoring co-twins (mean difference was 0.08).

Although paired-sample tests account by design for shared confounders, individual-level confounders cannot be accounted for. Therefore, the associations reported in the aforementioned studies may not be indicative of a causal pathway from the exposure to the outcome if parameter γ is not 0 in model (5).

### 3.6. GEE Models

A GEE model can be expressed as:

(7) $$g\left[E\left(Y_{ij}\right)\right]=\mu +\beta x_{ij}+\gamma z_{ij},$$

where $Y_{1j}$ and $Y_{2j}$ are assumed to be correlated for each j, and the unknown correlation is to be estimated. Since there are no pair effects, Equation (7) is a marginal rather than a conditional model, as all the previous ones were [48].

Using GEE models in children aged 9 or 12 years, Gong et al. [37] found that ADHD and autism spectrum disorders were not significantly associated with exposure to PM_10_ and NO_x_ during pregnancy, and during the first/ninth year of life. In children aged 18–22 months, Boghossian et al. [36] found that antenatal corticosteroids (ANS) were associated with a lower risk of neurodevelopmental impairment (NDI) or death among non-small for gestational age infants (RR = 0.89), and a higher risk among small for gestational age infants (RR = 1.62). Moreover, ANS were associated with a higher risk of NDI/death among infants of mothers with diabetes (RR = 1.55). In children aged 3–7 years, Johnson et al. [38] found positive associations between BMI and the frequency of maternal non-verbal encouragements/discouragements and temporary discouragements during a laboratory meal. Using GEE models, Yeung et al. [26] and Slob et al. [30] found similar results than in their conditional analyses (GLMMs and conditional logistic regression, respectively).

Since Equation (7) is a marginal model, parameter β represents a population-averaged effect. However, GEE models yield essentially the same estimates of β as in GLMMs with identity link function and in log-linear models. Conversely, studies using logistic regression [26,30,37] may have provided attenuated estimates of the within-pair effects (see Discussion). When several confounders are included in the model (as in all the aforementioned studies), the degree of attenuation decreases, and the reported associations may be considered indicative of a causal pathway.

### 3.7. GLMs with Cluster-Robust Standard Errors

A GLM with cluster-robust standard errors can be expressed as model (7), but no correlation parameter is estimated. Since ignoring pairwise dependence induces a bias in the standard error estimates, these are corrected through sandwich estimators [49]. Using this approach in children aged 4 years, Petkovsek et al. [41] found that, in females, the effect of prenatal smoking on externalizing behavioral problems was stronger in children with higher genetic risk (β for interaction =0.36). In twins aged 12–23 years, Palmer et al. [40] found that prenatal smoking significantly increased the frequency of conduct disorders (β=0.43) and inattention (β=0.85). In children aged 4–7 years, Jackson et al. [24] found that the effect of a short duration of breastfeeding on conduct problems was stronger in children with higher genetic risk (β for interaction =0.095). Örtqvist et al. [29] found that fetal growth was associated with post-bronchodilator FEV_1_ (β=−0.16) and abnormal FEV_1_ (OR = 1.27). In children aged 9–12 years, Castellheim et al. [39] found that lifetime general anesthesia was significantly associated with ADHD scores (β=1.02). Finally, Leong et al. [31] found that antibiotic use was associated with increasing BMI (β=0.018) and obesity (OR = 1.09) in children aged 4 years.

Since pair effects were not included in these models, shared confounders were not adjusted for by design. Therefore, despite the inclusion of several covariates, the effects sizes reported in the aforementioned studies may not be considered indicative of a causal pathway from the exposures of interest to the outcomes. Of note, Leong et al. [31] found that the reported associations disappeared after including pair effects in the model.

### 3.8. GLMs

A plain GLM can be expressed as model (7), but neither correlation parameters nor cluster-robust standard errors are provided. Using this approach in children aged 6 years, González-Valenzuela et al. [42] found that cesarean birth was associated with verbal (OR = 2.83), non-verbal (OR = 2.55), and global (OR = 3.69) delays, and with general intelligence difficulties (OR = 2.62). Although several covariates were included in the models, the reported associations may not be considered indicative of a causal pathway from the exposure to the outcome.

### 3.9. Independent-Sample Tests

Using independent-sample tests (Chi-square tests) in children aged 2–10 years, Zafman et al. [43] found that delivery order (length of exposure to the maternal vaginal–fecal microbiome) was not associated with asthma and obesity indicators. Since confounding effects were totally disregarded in this case, the reported associations should not be considered indicative of a causal pathway from the exposure to the outcome.

## 4. Discussion

We reviewed the statistical approaches used in twin studies relating exposure to childhood obesity, allergic, and neurodevelopmental disorders. We identified 32 studies published between 2011 and 2021, and nine classes of statistical approaches. Five were conditional approaches (within-pair analyses), while four were marginal approaches (unpaired analyses). We provided a description of the identified statistical methodologies, highlighting the differences and connections among them.

Twinning was disregarded in two studies [42,43], where independent-sample tests and a plain GLM were applied. In this regard, ignoring the correlation between observations leads to sub-optimal effect-size estimators (they are not the best linear unbiased estimators–BLUE–in linear regression) and biased standard error estimators, invalidating inference [50]. In particular, this leads to overestimating parameter uncertainty for individual-level covariates and to underestimating parameter uncertainty for shared covariates [51]. Moreover, unless many confounders are included, these models may not be appropriate to unveil causal relationships between the exposures and outcomes.

Six studies [24,29,31,39,40,41] adopted a “corrective” approach by adjusting the standard errors through sandwich estimators. In this case, however, the effect-size estimators remain sub-optimal. Conversely, five studies [26,30,36,37,38] adopted a “preventive” approach, i.e., the GEE model. In the absence of unmeasured or disregarded confounding, GEE models provide optimal effect-size estimators (they are BLUE in linear regression), and correct standard error estimators [9,48].

Whereas all the aforementioned approaches are “marginal”, all the other identified approaches are “conditional”, since their parameters represent outcome differences associated with an exposure difference within a twin pair [4,5]. Consequently, conditional models adjust by design for shared confounders, i.e., the genome and the twin-identical exposome, and may be more appropriate to unveil causal relationships.

Two studies [33,35] applied the simplest form of conditional analysis, i.e., paired-sample tests; however, this approach provides no way to adjust for individual-level confounders. Conversely, three studies [32,33,34] carried out within-pair difference analyses, which have the potential to adjust for individual-level confounders by the inclusion of additional difference terms in the model. However, only Bogl et al. [32] included the difference in an individual-level confounder (height) in their model, while simple correlation coefficients were used in the other two studies [33,34]. Within-pair difference models cannot be applied if the link function is different from the identity.

GLMs with fixed pair effects can be applied in more general settings. This model was applied to a quantitative outcome in two studies [29,31] as “a way to control for a greater amount of unmeasured confounding” [31] compared to linear regression with robust standard errors. Only in one study [31], model (4) was applied to a binary outcome (logistic regression) using dummy variables for the pair effect. However, in this case, the estimator of β is severely biased, and using conditional logistic regression is much more recommended [52]. Conditional logistic regression was, indeed, used in three studies [28,29,30].

Although GLMs with fixed pair effects adjust by design for shared confounders, the associated parameters are not identifiable if included in the model. Conversely, GLMMs can incorporate shared covariates, simply leading to a lower heterogeneity captured by the random intercept [48]. As a result, estimates of β from GLMMs are quite robust with respect to the omission of shared covariates [48]. Using a GEE rather than a GLMM approach leads essentially to the same estimate of β in models with identity link function and in log-linear models [9,48]. Conversely, logistic regression estimated through the GEE approach generally yields attenuated estimates of β, and the degree of attenuation increases when shared covariates are omitted [48]. Five studies applied a GLMM [23,24,25,26,27]; of note, all but one [17] of the studies including repeated measurements of the outcome applied a GLMM [23,25,26,27].

Finally, ACE models were applied by eleven studies [12,13,14,15,16,17,18,19,20,21,22]. These models keep the advantages of all the previous models, but in addition, they can disentangle the contribution of the genome and the exposome. However, such models may appear difficult to implement and to interpret, and their extension to longitudinal data through mixed-effect modelling is not straightforward [53]. In this review, only one study [17] applied an ACE model to repeated measurements, using its bivariate extension. In ACE models, genetics is often the exposure of interest, while shared and individual covariates are incorporated into C and E, respectively. In this review, covariates were included in the ACE model only in one study [12], while they were regressed out prior to analyses (or considered as potential moderators) in almost all the other studies. Table 1 summarizes the strengths and limitations of the aforementioned methods and dedicated libraries for implementation in the R software.

**Figure 2:**
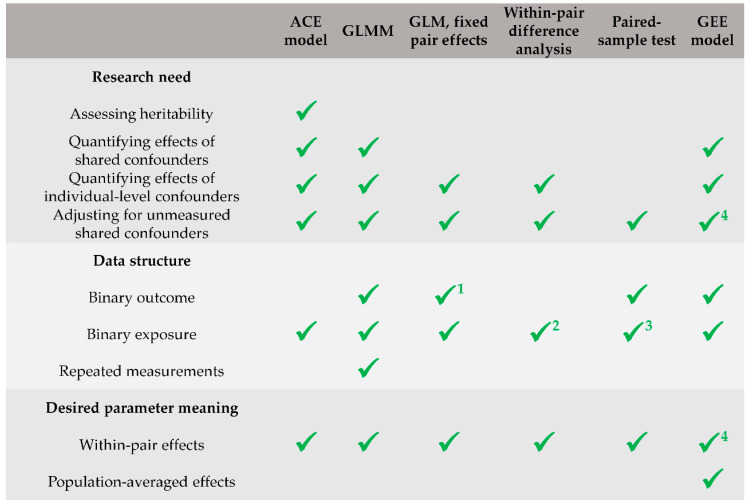
Summary of recommendations regarding which types of models researchers should favor under different conditions (research needs, data structure, and desired parameter meaning). ^1^ Conditional logistic regression is recommended. ^2^ Accommodated for through the use of dummy variables. ^3^ Either the outcome or the exposure mustable t be binary. ^4^ Only in models with identity link function and in log-linear models.

In practical applications, the best (or sufficient) approach to use may depend on the research needs, data structure, and desired parameter meaning. ACE models can accommodate for most of the research needs, including the assessment of heritability, but they require adaptations for binary outcomes and repeated measurements. GLMMs are a valid choice if assessing heritability is not a research need, and they would be the best choice to accommodate for binary outcomes and repeated measurements. GLMs with fixed pair effects and within-pair difference analyses are good (and substantially equivalent) approaches when the effects of shared confounders do not need to be explicitly quantified, but only the former can accommodate for binary outcomes (conditional logistic regression is recommended). If either the outcome or the exposure is binary, and confounding effects are assumed to be marginal (e.g., if the twins are substantially identical except that for the exposure of interest), paired-sample tests can be a valid alternative to more complex models. The use of GEE models (marginal models) for logistic regression should only be limited to situations in which the researchers are interested in estimating population-averaged effects rather than within-pair effects. In these cases, however, the twin design is not strictly needed, and is not more useful than a singleton study (except that for increasing the sample size). Finally, GLMs should never be used in a twin study, since they may lead to sub-optimal inference. Figure 2 provides a summary of recommendations regarding which types of models researchers should favor under different conditions (research needs, data structure, and desired parameter meaning).

Among other methods for twin data, DeFries–Fulker regression was only mentioned by Petkovsek et al. [41]. ADE models were only mentioned by Faith et al. [13]. ACE models accounting for correlated errors and “twin confusion” were only mentioned by Lamb et al. [16]. ACDE models [44] and ACE models for categorical outcomes [54] were never mentioned. Incomplete records were generally excluded, and the issue of missing value imputation appears to have been only addressed in one study [26]. Indeed, imputation methods for correlated data might be less popular than standard approaches; in general, likelihood approaches (such as GLMM) have shown to be robust to the “missing completely at random” assumption, while non-likelihood marginal models (such as GEE models) have not [55].

Twin studies also have disadvantages. First, generalizability to singleton populations may not always be possible [56]. In this regard, twins may differ from singletons in several aspects: twins tend to have older parents, to be born preterm, and to have lower birthweight and specific disorders. Moreover, as pointed out by Bilenberg et al. [35], the genetic control may reduce the exposure contrast, and larger sample sizes may be required. However, due to the relative rarity of twins, gathering a large sample size may not be easy. Finally, experimental trials involving twins are statistically inefficient if all the twins in the same pair are allocated to the same treatment arm (the treatment becomes a shared covariate, leading to large standard errors) [8].

This review has some limitations that should be acknowledged. Study outcomes in the search strategy were limited to obesity, asthma, eczema, rhinitis, and neurodevelopmental disorders. Therefore, the distribution of statistical methods captured in this review may not generalize to the broader twin literature. Moreover, although we have used the general keyword “expos*” in the search strategy, we limited to search this term in titles and abstracts; therefore, as with all reviews, we might not have identified all the relevant articles. Finally, the characteristics of the included studies were sometimes difficult to identify and to extract (e.g., the number of twin pairs by zygosity) and, in some cases, they were inferred by the reviewers (e.g., based on tables).

## 5. Conclusions

In conclusion, twin data provide a unique opportunity to control for confounding and disentangling the role of the human genome and exposome when investigating disease etiology. Although rare, sub-optimal (GLMs with fixed pair effects for binary outcomes) or simplistic (plain GLMs and independent-sample tests) approaches were still present in the twin-study literature, possibly undermining the validity of the evidence base. By highlighting the strengths and limitations of commonly applied statistical methods, this review may be helpful for researchers using twin designs.

## Figures and Tables

**Table 1:** Classes of statistical methods used in the reviewed studies.

Class of Methods	Strengths	Limitations	R Libraries
ACE models	- Confounders can be included - Optimal inference - Shared confounders are adjusted for by design - Genetic contribution can be estimated	- Require adaptations for binary outcomes and repeated measurements	umx lavaan OpenMx
Generalized linear mixed models (GLMMs)	- Suitable for binary outcomes - Confounders can be included - Optimal inference - Shared confounders are adjusted for by design - Suitable for repeated measurements	- Genetic contribution cannot be estimated	lme4 nlme MASS
GLMs with fixed pair effects	- Suitable for binary outcomes - Individual-level confounders can be included - Shared confounders are adjusted for by design	- Shared confounders cannot be included - Estimators may be sub-optimal - Unsuitable for repeated measurements - Genetic contribution cannot be estimated	stats
Within-pair difference analyses	- Individual-level confounders can be included - Optimal inference - Shared confounders are adjusted for by design	- Unsuitable for binary outcomes - Shared confounders cannot be included - Unsuitable for repeated measurements - Genetic contribution cannot be estimated	stats
Paired-sample tests	- Optimal inference - Shared confounders are adjusted for by design	- Require a binary exposure - Require adaptations for binary outcomes - Confounders cannot be included - Unsuitable for repeated measurements - Genetic contribution cannot be estimated	stats
Generalized estimating equations (GEE) models	- Suitable for binary outcomes - Confounders can be included - Optimal inference	- Shared confounders are not adjusted for by design - Require adaptations for repeated measurements - Genetic contribution cannot be estimated	gee geepack
Generalized linear models (GLMs) with cluster-robust standard errors	- Suitable for binary outcomes - Confounders can be included - Optimal standard error estimators	- Sub-optimal effect-size estimators - Shared confounders are not adjusted for by design - Unsuitable for repeated measurements - Genetic contribution cannot be estimated	sandwich
Generalized linear models (GLMs)	- Suitable for binary outcomes - Confounders can be included	- Sub-optimal inference - Shared confounders are not adjusted for by design - Unsuitable for repeated measurements - Genetic contribution cannot be estimated	stats
Independent-sample tests	- Suitable for binary outcomes	- Require a binary exposure - Confounders cannot be included - Sub-optimal inference - Shared confounders are not adjusted for by design - Unsuitable for repeated measurements - Genetic contribution cannot be estimated	stats

## Supplementary Materials

The following are available online at https://www.mdpi.com/article/10.3390/ijerph182312696/s1. Supplementary Material S1: Characteristics of the included studies. Search strategy: “A critical review of statistical methods for twin studies relating exposure to early life health conditions: Search Strategy”, Table S1: Characteristics of the included studies.
