# Supplementary material for: A Critical Review of Statistical Methods for Twin Studies Relating Exposure to Early Life Health Conditions
Source: Int J Environ Res Public Health. 2021 Dec 2;18(23):12696. doi: 10.3390/ijerph182312696 (PMC8657152; doi:10.3390/ijerph182312696)
Supplement: Supplementary file 1 [file ijerph-18-12696-s001.zip › ijerph-1456465_Supplementary Material S1 .pdf]

Supplementary Material S1: A critical review of statistical methods for twin studies relating exposure to early life health conditions: Search Strategy

PUBMED

(“Infant”[Mesh] OR “Child”[Mesh] OR newborn*[TIAB] OR infant*[TIAB] OR child*[TIAB]) AND (“Twins”[Mesh] OR twin*[TIAB]) AND (“Diet, Food, and Nutrition”[Mesh] OR “dietary habit*”[TIAB] OR “dietary behav*”[TIAB] OR “food habit*”[TIAB] OR “food behav*”[TIAB] OR “nutrition habit*”[TIAB] OR “nutrition behav*”[TIAB] OR “nutritional habit*”[TIAB] OR “nutritional behav*”[TIAB] OR “eating habit*”[TIAB] OR “eating behav*”[TIAB] OR “feeding habit*”[TIAB] OR “feeding behav*”[TIAB] OR “alimentary habit*”[TIAB] OR “alimentary behav*”[TIAB] OR expos*[TIAB]) AND (“Asthma”[Mesh] OR asthma*[TIAB] OR “Eczema”[Mesh] OR eczema[TIAB] OR “atopic dermatitis”[TIAB] OR “Rhinitis”[Mesh] OR rhinitis[TIAB] OR “Obesity”[Mesh] OR obes*[TIAB] OR “Neurodevelopmental Disorders”[Mesh] OR neurodevelopment*[TIAB] OR neuropsycholog*[TIAB]) AND y_10[Filter] AND english[Filter] NOT (“animals”[Mesh] NOT “humans”[Mesh])

SCOPUS

TITLE-ABS-KEY(infant* OR newborn* OR child*) AND TITLE-ABS-KEY(twin*) AND TITLE-ABS-KEY(“dietary habit*” OR “dietary behav*” OR “food habit*” OR “food behav*” OR “nutrition* habit*” OR “nutrition* behav*” OR “eating habit*” OR “eating behav*” OR “feeding habit*” OR “feeding behav*” OR “alimentary habit*” OR “alimentary behav*” OR expos*) AND TITLE-ABS-KEY(asthm* OR eczema OR “atopic dermatitis” OR rhinitis OR obes* OR neurodevelopment* OR neuropsycholog*) AND PUBYEAR AFT 2010 AND LANGUAGE(english)

WEB OF SCIENCE

(TI=(infant* OR newborn* OR child*) OR AB=(infant* OR newborn* OR child*)) AND (TI=twin* OR AB=twin*) AND (TI=(“dietary habit*” OR “dietary behav*” OR “food habit*” OR “food behav*” OR “nutrition* habit*” OR “nutrition* behav*” OR “eating habit*” OR “eating behav*” OR “feeding habit*” OR “feeding behav*” OR “alimentary habit*” OR “alimentary behav*” OR expos*) OR AB=(“dietary habit*” OR “dietary behav*” OR “food habit*” OR “food behav*” OR “nutrition* habit*” OR “nutrition* behav*” OR “eating habit*” OR “eating behav*” OR “feeding habit*” OR “feeding behav*” OR “alimentary habit*” OR “alimentary behav*” OR expos*)) AND (TI=(asthm* OR eczema OR “atopic dermatitis” OR rhinitis OR obes* OR neurodevelopment* OR neuropsycholog*) OR AB=(asthm* OR eczema OR “atopic dermatitis” OR rhinitis OR obes* OR neurodevelopment* OR neuropsycholog*)) AND PY=(2011-2021) AND LA=(English)

EMBASE

('infant'/exp OR 'child'/exp OR 'newborn'/exp OR newborn*:ab,ti OR infant*:ab,ti OR child*:ab,ti) AND ('twins'/exp OR twin*:ab,ti) AND ('diet'/exp OR 'food'/exp OR 'nutrition'/exp OR 'dietary habit*':ab,ti OR 'dietary behav*':ab,ti OR 'food habit*':ab,ti OR 'food behav*':ab,ti OR 'nutrition habit*':ab,ti OR 'nutrition behav*':ab,ti OR 'nutritional habit*':ab,ti OR 'nutritional behav*':ab,ti OR 'eating habit*':ab,ti OR 'eating behav*':ab,ti OR 'feeding habit*':ab,ti OR 'feeding behav*':ab,ti OR 'alimentary habit*':ab,ti OR 'alimentary behav*':ab,ti OR expos*:ab,ti) AND ('asthma'/exp OR asthma*:ab,ti OR 'eczema'/exp OR eczema:ab,ti OR 'atopic dermatitis':ab,ti OR 'rhinitis'/exp OR rhinitis:ab,ti OR 'obesity'/exp OR obes*:ab,ti OR 'neurodevelopmental impairment'/exp OR neurodevelopment*:ab,ti OR neuropsycholog*:ab,ti) AND [english]/lim AND [embase]/lim AND [2011-2021]/py NOT ([animals]/lim NOT [humans]/lim)
